# A Framework and Algorithm for Human-Robot Collaboration Based on Multimodal Reinforcement Learning

**DOI:** 10.1155/2022/2341898

**Published:** 2022-09-28

**Authors:** Zeyuan Cai, Zhiquan Feng, Liran Zhou, Changsheng Ai, Haiyan Shao, Xiaohui Yang

**Affiliations:** ^1^School of Information Science and Engineering, University of Jinan, Jinan 250022, China; ^2^Shandong Provincial Key Laboratory of Network Based Intelligent Computing, University of Jinan, Jinan 250022, China; ^3^School of Mechanical Engineering, University of Jinan, Jinan 250022, China; ^4^State Key Laboratory of High-end Server & Storage Technology, Jinan, China

## Abstract

Despite the emergence of various human-robot collaboration frameworks, most are not sufficiently flexible to adapt to users with different habits. In this article, a Multimodal Reinforcement Learning Human-Robot Collaboration (MRLC) framework is proposed. It integrates reinforcement learning into human-robot collaboration and continuously adapts to the user's habits in the process of collaboration with the user to achieve the effect of human-robot cointegration. With the user's multimodal features as states, the MRLC framework collects the user's speech through natural language processing and employs it to determine the reward of the actions made by the robot. Our experiments demonstrate that the MRLC framework can adapt to the user's habits after repeated learning and better understand the user's intention compared to traditional solutions.

## 1. Introduction

The limitations of “human-centered” or “robot-centered” robots have become increasingly essential with the advancement of the theories and applications of natural human-robot interaction. Regardless of the type of robots, understanding human intentions is indispensable for achieving complex human-robot collaboration processes [1]. A well-designed robot should dynamically adapt its behavior to different tasks and help humans more efficiently and respond to changes in the environment, humans, and own state in real time. In our daily life, we often communicate with others by expressing behavioral intentions [2–4] (such as gazes, gestures, and actions) to obtain a tacit understanding when collaborating with them. These intentions are frequently expressed through messages such as body language, voice, and mannerisms.

Perceiving the information mentioned above is not a complex problem for robots, and there have been many related studies making robots more effective in perceiving people and environments [5]. However, this modal information obtained through sensors does not intuitively and accurately express real human intentions. Using this information rationally to acquire the exact human intentions is a popular research field at present.

## 2. Related Work

### 2.1. Human-Robot Collaboration and Intention Understanding

Many excellent algorithms have emerged in implementing human-robot collaboration based on intention understanding. In 2007, Suzuki et al. [6] estimated the intention of a paraplegic patient using the reaction forces on the floor while walking and standing. Their conclusions demonstrated the effectiveness of this approach in supporting the daily life of people with disabilities. In 2013, the Bayesian model was applied by Wang et al. [7] to estimate intentions on kinetic models generated in the process of human motion and recognition intention results were updated when additional motion data were obtained. In 2015, Ref. [8] employed manually applied forces and hip rotations to identify motion intention. Their conclusions verified that the method was effective in helping older people with turning assistance. In 2018, a hidden Markov model was used by Berg et al. [9] to recognize human motion, allowing robots to better adapt to human behavior and achieve human-robot synergy on production lines. Reference [10] utilized brain-computer interfaces (BCIs), visual interfaces, and remote robots to perform “emulated haptic shared control,” through which a remote proximity perception system was established to achieve human-robot collaboration and enable tetraplegic patients to interact with friends and the environment. Reference [11] performed excellent work in the opposite direction by proposing a method to interpret robot behavior as intention signals using natural language sentences, so as to better reveal robot behaviors and reduce misunderstandings caused by information asymmetry. Reference [12] proposed the proactive incremental learning (PIL) framework that learned the connection between human gestures and robot actions, which contributes to efficient human-robot interaction. In 2019, Ref. [13] proposed a cooperative fuzzy impedance control with embedded safety rules, a method that provides assistance and safety for human operators in heavy industrial scenarios. Reference [14] computed the probability distribution of intentions corresponding to each modality and then output these distributions through a Bayesian approach to combine independent opinion bases. The results suggested that this approach was better in accuracy and robustness than a unimodal-based classifier. In 2021, Ref. [15] achieved intention understanding using a single modality, gaze. Their study confirmed that gaze enabled more efficient human-computer collaboration in specific scenarios. In addition to conventional modalities (such as gesture, gaze, voice, and action), many studies exploited the information of less detectable modalities for intention understanding. For example, Ref. [16] designed a new approach for intention understanding of upper limb movements using mobile electroencephalography (EEG) via LSTM-RNN, which could provide early warning of impending danger to improve the safety of the system. Reference [17] constructed a projective recurrent neural network to estimate the joint angular intention of the user during motion using a Hill-based muscle model. Another interesting approach exploits the human's preference to adapt the robot's behavior based on the human's feedback. Reference [18] discussed the necessity of user preferences in the design of robotic exoskeletons. Reference [19] proposed a path-based velocity planner, which uses the optimization method based on user pairwise preferences, can classify different paths and adjust the robot execution velocity more finely.

### 2.2. Reinforcement Learning

Reinforcement learning, as a field of artificial intelligence, has made significant progress since its introduction. An increasing number of human-robot collaborations use reinforcement learning to handle their problems. Reference [20] proposed the DQN algorithm in 2015. The core idea of the DQN algorithm is to use the strong fitting property of neural networks to calculate the score of each action A in the input state S. It tackles the problem that the Q-Learning algorithm cannot handle scenarios with large state spaces. DQN takes two neural network structures of the same architecture: Q_target and Q_eval. The former intermittently updates the parameter *θ*^−^, while the latter updates the parameter *θ* in real time. Additionally, DQN experience replay saves the experienced state-action pairs (s, a), the corresponding reward, and next states in a memory bank, from which previous experiences randomly selected from the memory bank can be learned while the DQN is iterating. DDQN [21] is an improved version of DQN. Although DQN has been revealed to be effective in many applications, it still has some shortcomings. Since DQN uses the maximum operation to estimate the reward for the next state, DQN overestimates the Q value after several iterations. DQN strips the selection of the action and the evaluation of the action, estimates the best action in the Q_eval network parameters, and finds the score of the action in the Q_target network parameters. The authors put forward theoretical and experimental suggestions that DDQN effectively eliminates the drawbacks of DQN overestimation.

In 2019, Ref. [22] adopted the Soft Actor Critic reinforcement learning algorithm to build a robotic platform that the robot is capable of learning cooperative tasks with people in only 30 minutes without simulation training. Reference [23] proposed a multirobot path planning algorithm with deep q-learning combined with a convolutional neural network algorithm. Their simulation results revealed that the robot using this method had flexible and efficient motion performance in various environments compared to the traditional method. Reference [24] first applied deep reinforcement learning to the Urban Search and Rescue Team. They combined deep reinforcement learning with the robotic exploration of uncharted territory, which allowed the robot to explore the location environment autonomously. Their experiments demonstrated that the method could shrink victims faster than other methods. Inspired by Google Deep Mind, Ref. [25] formatted the collaborative human-robot assembly workflow as a chessboard. Specifically, the motion selection on the chessboard was used to simulate the human and robot decision-making in the human-robot collaborative assembly workflow. The self-training algorithm based on reinforcement learning was used for training, and the best strategy of collaborative human-robot work sequence was obtained without guidance or domain knowledge beyond the rules of the game, so as to improve efficiency. Reference [26] encoded the task and safety-related requirements into reinforcement learning and applied reinforcement learning to protect users in the process of human-robot cooperation. Reference [27] designed a human-centered collaborative system based on reinforcement learning that adopted unsupervised end-to-end learning to effectively tackle uncertainty in human behavior recognition and improve the behavioral decisions of the robot. In this way, the risks and benefits achieved by the robot after taking action were balanced. In 2020, Ref. [28] modeled the complex human-computer interaction dynamics and proposed a model-based reinforcement learning variable impedance control, which minimizes the human force consumption. In 2021, Ref. [29] used reinforcement learning on dynamic task partitioning in assembly tasks with good results. Reference [30] presented an adaptive training method based on a deep Q-learning approach. The method treated robots and humans as agents in an interactive training environment for learning. Their algorithm addressed how to consider the dynamics of the time dimension and the stochasticity in the program sequence when the collaborative assembly was an industrial task.

Reinforcement learning has demonstrated its powerful role in robotics by enabling robots to learn independently [31]. In summary, human-robot collaboration cannot be separated from the process of intention understanding. The fusion of multiple modalities has been discovered to have higher robustness in the correctness of intention understanding. Most human-robot collaboration frameworks based on intention understanding have explored an intention understanding paradigm working for all users to improve the robustness of the algorithm and the accuracy of intention understanding. However, it is tough to find a suitable paradigm for all users, as each user has different habits in expressing their intentions due to the variability among individuals. It is a problem that needs to be solved to make the robots work efficiently with various users.

To this end, a multimodal human-robot collaboration (MRLC) framework based on reinforcement learning is proposed in this article. MRLC is divided into two parts. The first part is the intention understanding process based on the deep reinforcement learning algorithm (DDQN) [21], which adapts to the habits of individuals through iterative training and forms habit rules for each user. Hence, our collaboration framework can eliminate the issues of inconsistent collaboration when facing users with different habits in traditional human-robot collaboration. The second part is the task assignment process. Through the first part of intention understanding, the robot understands “what the user wants to do” and then MRLC assigns tasks to the robot to collaborate with the user.

This article has three main contributions:

1. The MRLC human-robot collaboration framework is proposed. Reinforcement learning is adopted for intention recognition to make the human-robot collaboration framework more robust to different users.
2. The MRLC framework uses a reward function incorporating natural language processing, which maintains the user experience during robot's learning process.
3. The MRLC human-robot collaboration framework is successfully applied to a scenario where humans and robots work together to build a Jenga tower.

A human-robot collaboration scenario is designed for building the Jenga tower to verify the feasibility of our proposed collaboration framework. In this scenario, the robot needs to consider the state of the Jenga tower and user's intention to achieve dynamic collaboration among the robot, Jenga tower, and user.

## 3. Materials and Methods

### 3.1. MRLC Structure

**Figure 1:**
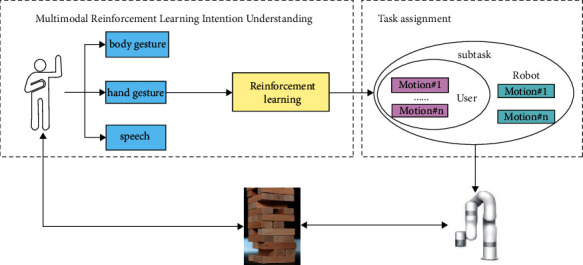
The structure of MRLC.

Existing human-robot collaboration frameworks mainly use a unified paradigm to observe user's characteristics to achieve intention understanding and human-robot collaboration. In contrast, the MRLC framework has the following features. (1) MRLC is capable of adapting to each user's behavioral habits rather than applying a uniform paradigm to every user. (2) It enables a three-way interaction between robot and user, user and environment, and robot and environment. The structure of MRLC is illustrated in Figure 1. The main goal of MRLC is to allow the robot to learn user's behavioral habits, to recognize user's intentions, and to assign tasks to the user's following different intentions. It guarantees that the robot can perform human-robot collaboration tasks dynamically and safely.

The MRLC framework is divided into two main modules: multimodal intention understanding module and task assignment module. First, the robot collects the characteristics of user's three modalities and learns user's behavioral habits through reinforcement learning to obtain user's behavioral intention. After user's intention is obtained, the robot enters the task assignment phase, in which the robot's action sequence is specified based on user's behavior. Then, the robot starts to interact with the environment and the user.

### 3.2. Multimodal Intention Understanding Based on Reinforcement Learning

The MRLC framework contains a novel multimodal reinforcement learning intention understanding algorithm. The core idea is to learn users' behavioral habits through deep reinforcement learning in iterative iterations, so as to eliminate errors induced by differences in behavioral habits of different users and to achieve a more robust intention understanding.

**Figure 2:**
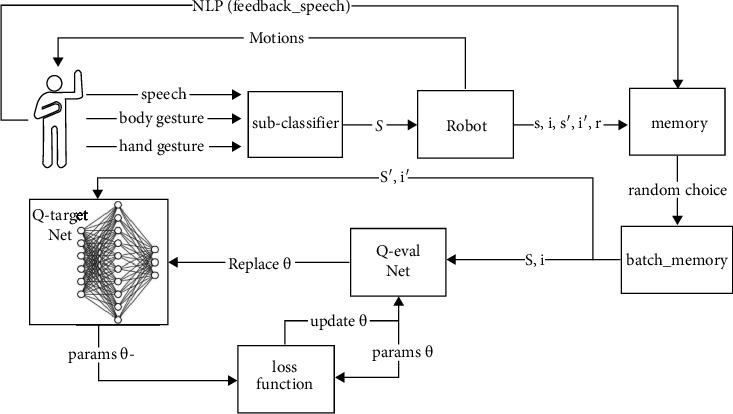
Multimodal reinforcement learning intention understanding algorithm architecture. **s** represents the user's features, **s**′ represents the user's features next time, *i* represents the result of intention understanding, and *i*′ represents the result of intention understanding next time.

Figure 2 illustrates the architecture of the multimodal reinforcement learning intention understanding algorithm. It is divided into three stages: (1) extraction of user multimodal features. The data obtained from the sensors first go through three subclassifiers to obtain the classification results of *m*1, *m*2, and *m*3. The user modal data are finally converted into a 3D vector **s** = [*m*1, *m*2, *m*3]; (2) the extracted user features are used as state inputs to fit the scores under each intention outcome; (3) calculation of the optimal operation corresponding to the user intention according to the optimization objective by equation (1), followed by the analysis of the user's linguistic feedback using NLP to obtain the user satisfaction *S*_*a*_, which is learned iteratively as part of the reward:

(1) $$\begin{array}{c} i=\max Q\left(s,I;\theta \right), \end{array}$$

where **s** represents the user's features, *i* represents the best intention at moment, **I** represents intentions spaces, *Q* represents the value of each intention calculated using the q_eval neural network, and *θ* represents the parameter of the q_eval neural network:

### 3.3. User Multimodal Feature Extraction

The user's behavioral characteristics are defined as autonomous, natural movements (such as gestures and speeches) made during human-robot collaboration.

**Figure 3:**
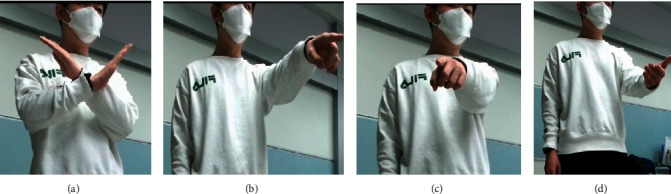
User's body gesture. (a) Cross arms; (b) point to the unplaced pile of blocks; (c) point to the built Jenga tower; (d) raise your hand in a small increment.

**Figure 4:**
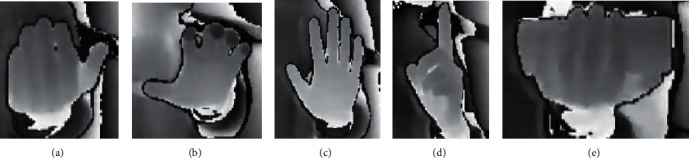
User's hand gestures. (a) Fingers bent slightly upward; (b) fingers bent slightly downward; (c) open palm; (d) index finger up; (e) pick up a block.

Three sensors are arranged to implement the user's input in three modalities: speech, body gesture, and hand gesture. In our work, a stable and efficient data processing method is selected to process the data obtained from the sensors. Regarding speech modality, the user's speeches are converted into text and classified into seven categories by combining keyword recognition. Another interesting approach is the use of pointing to achieve natural human-computer interaction. There has been a lot of outstanding work [32, 33] on their methods to detect user pointing accurately. However, considering the complexity of the system, we do not apply these results in our system. The category numbers corresponding to the speech keywords are listed in Table 1. Concerning body gesture modality, KinectGesture in KinectV2 is adopted to implement the recognition of four types of static user body gestures. With respect to hand gesture modalities, efficientnetV2 is employed to implement five types of hand gesture recognition. All the body gestures and hand gestures that can be detected are exhibited in Figures 3 and 4.

### 3.4. Reward Functions Based on Natural Language Processing

In the process of human-robot collaboration, it is tricky to determine an appropriate reward function while ensuring the user experience. The robot needs to know whether its behavior is correct during the learning process. Telling the robot whether the behavior is correct every time by manual input would cut off the user experience.

Therefore, natural language processing techniques are utilized to collect users' evaluations: NLP (feedback_speeches). With the snownlp module, speech emotion analysis is performed on the speech collected by the microphone. The result of NLP (feedback_speeches) is between 0 and 1 and is judged as positive feedback when the result is higher than 0.5. The intention is understood correctly when the user's evaluation is positive (such as “good job”). Notably, if the user does not give any feedback, we believe the user's tacit approval of the behavior and regard it as positive feedback:

(2) $$\begin{array}{c} R=\left\{\begin{array}{cc} 10, & \text{NLP}\left(\text{feedback\_speeches}\right)>0.5\text{ or nonexistent,}\\ 10, & \text{NLP}\left(\text{feedback}\_\text{speeches}\right)<0.5. \end{array}\right) \end{array}$$

### 3.5. Multimodal Reinforcement Learning Intention Understanding Algorithm Network Structure

The multimodal reinforcement learning intention understanding algorithm proposed in this article has two neural networks with the same structure: q_target and q_eval [21], both of which consist of two fully connected layers *l*1 and *l*2. Among them, *l*1 consists of 50 neurons. The multimodal reinforcement learning intention understanding algorithm also adopts a memory bank to store the previously learned results and implement the offline learning. The input to q_eval is the user features *s*, which are also the results of the three subclassifiers. q_eval first fits the user features *s* using random weights to derive a score for each state. The intention with the highest score is selected as the best result for output. After the user gives feedback, the reward value *R* is derived according to the reward function (2). Then, the sum of current rewards and expected future rewards y' is calculated by the following equation:

(3) $$\begin{array}{c} y^{\prime }=R+\gamma *\max_{\;i}Q\left(s^{\prime },\max_{\;i}Q\left(s^{\prime },I;\theta \right)\right);\theta^{-}, \end{array}$$

where *γ* represents the decay factor of future reward, *θ* represents the parameter of q_eval net, *θ*^−^ represents the parameter of q_target net, *R* represents the current reward, **s**' designates the multimodal input at the next intention understanding, and **I** represents the intentions spaces.

Since the update frequencies of q_target and q_eval are different, the following loss function can be obtained by using Temporal-Difference (TD).

(4) $$\begin{array}{c} \text{Loss}={\left(y^{\prime }-Q\left(s,i;\theta \right)\right)}^{2}, \end{array}$$

where **s** denotes the multimodal input and *i* represents the result of intention understanding.

### 3.6. Task Assignment

In the previous section, the user's intention has been derived based on the multimodal reinforcement learning intention understanding algorithm. In this section, task assignment is performed based on the intention.

**Figure 5:**
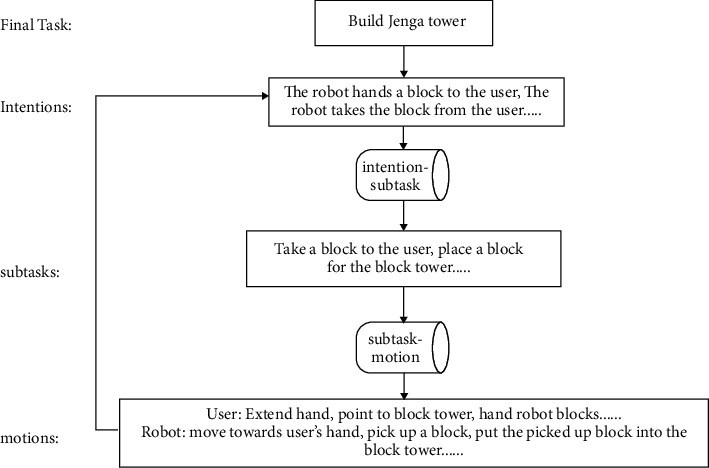
Task assignment process.

The MRLC framework uses a top-down, progressively refined dynamic task assignment approach as illustrated in Figure 5. Specifically, a database M(intention, task) of intentions and subtasks, and a database M(subtask, motion) of subtasks and actions are constructed. The final task is progressively refined to all pending action sequences Motion.

A reasonable task assignment module can dynamically assign tasks to the robot based on the user's behavior instead of rigidly specifying the tasks that the robot needs to be responsible for. Under the concept of sets in mathematics, all tasks are considered a full set Motion. The tasks that the user has completed are a subset Motion_user_. Then, the tasks that the robot needs to be responsible for are the complement of Motion_user_ where Motion_robot_ = Motion − Motion_user_. This approach allows the MRLC framework to achieve dynamic task assignments to further increase the flexibility of collaboration. Additionally, the MRLC framework can be easily applied to other collaboration scenarios by modifying the two databases.

For example, if Motion_user_ is {“user picks up a block”} and Motion is {“user picks up a block”, “robot moves towards user's hand”, “robot grabs the block in user's hand”}, then Motion_robot_  is  {“robot moves towards user's hand”, “robot grabs the block in user's hand”}.

### 3.7. Algorithm Analysis

Based on the above research ideas and the MRLC architecture diagram in Figure 1, a specific description of the MRLC architecture algorithm1 is presented as follows.

The MRLC framework aims to eliminate the bias in collaboration effectiveness caused by the variability of individual user habits. Multimodal reinforcement learning intention understanding methods are employed to learn each user's habits and thus weaken the impact of individual differences. One of the crucial metrics to evaluate the effectiveness of the MRLC framework is the correct rate of intention understanding, which is the core of the MRLC framework's ability to adapt to different user habits.

When a new user tries to collaborate with the robot for the first time in our human-robot collaboration scenario, the multimodal reinforcement learning intention understanding algorithm is set up to first sense the three modal information of the user and use it as input to predict the user's intention and perform task assignment; besides, the parameters of the algorithm are adjusted relying on the feedback given by the user; in this way, the issue of how to make the robot learn the user's habits is theoretically overcome. With an increase in the number of learning times, the multimodal reinforcement learning intention understanding algorithm gradually converges. The intention understanding becomes more effective, suggesting that the MRLC framework learns the user's habits.

The MRLC framework implements the perception of different modal data through an efficient subclassifier, instead of feeding the collected basic information directly into the deep reinforcement learning neural network to ensure real-time collaboration. The multimodal reinforcement learning intention understanding algorithm only needs to process a three-dimensional matrix representing multimodal information, which contributes to a significant decrease in the time complexity and ensure the real-time performance of the algorithm.

The MRLC framework theoretically addresses the issue presented in this article: how can robots still maintain efficient collaboration facing users with different habits?

## 4. Experimental Results and Analysis

### 4.1. Experimental Scenes

**Figure 6:**
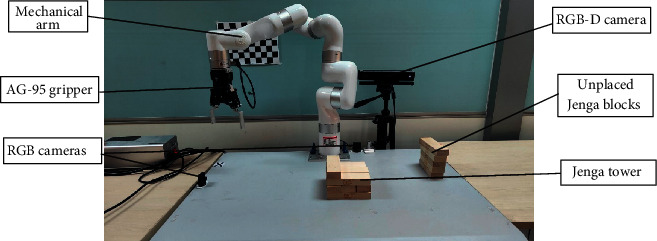
Schematic diagram of the experimental scene.

Our human-robot collaboration scenario is shown in Figure 6, which consists of the Xarm 7-axis mechanical arm, mechanical gripper AG-95, two RGB cameras, and an RGB-D camera, where two RGB cameras are used to detect the status of the Jenga tower. The computer's CPU, GPU, and RAM are I7–10875H, RTX2060, and 16G, respectively. Furthermore, microphones and an RGB-D camera are employed to capture the user's speeches, body gestures, and hand gestures.

### 4.2. Experimental Scenes

Ten experimenters, including six males and four females, with an average age of 25 years were invited to participate in the experiment. They had never been exposed to similar human-robot collaboration scenarios before. Before the experiment, we informed the experimenters about the modalities that the robot could perceive some specific modal data, such as specific hand gestures and keywords.

The task of human-robot collaboration is to establish Jenga tower rather than playing Jenga game. Six categories of intentions are set.  Table 2 lists all intentions and the corresponding numbers.

**Figure 7:**
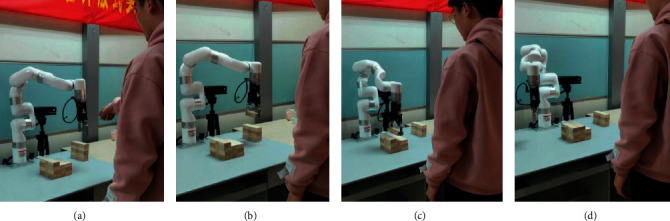
A typical human-robot collaboration process. (a) The user makes an act of pointing to an unplaced block and speaks the speech keyword “put the block on”; (b) the multimodal reinforcement learning intention understanding algorithm derives the user's intention: the robot actively picks up the blocks; (c) the user gives the speech evaluation: “That is it” NLP evaluates this feedback to be positive; (d) the robot is reset, and the reinforcement learning learns the user's habit and the round of collaboration ends.

A complete collaborative process is recorded, as exhibited in Figure 7. The robot and the user are in a face-to-face position, and the unplaced Jenga blocks are stacked on the side of the robot where the robot can easily clip them.

### 4.3. Experiment Procedure

#### 4.3.1. The Differences in User Habits When Expressing Intentions

It is necessary to demonstrate the differences in expression habits of different users when expressing the same intention. Thus, a questionnaire was distributed to the experimenters before the formal experiment to verify the specific modal categories used by each experimenter in expressing the same intention, in which we marked the specific body gestures, hand gestures, and keywords in Section 3.2.1. The table of the questionnaire is provided in Table 3.

#### 4.3.2. The Success Rate of Human-Robot Collaboration

In this section, the experiment is performed on the relationship between the success rate of human-robot collaboration and learning times, as well as the success rate of human-robot collaboration in the MRLC framework for new users with different habits.

To explore the relationship between the success rate of human-robot collaboration, ten people are divided into five groups: A1, A2, A3, A4, and A5; we limit the number of times the robot learns to 200, 400, 600, 800, and 1000 for the A1, A2, A3, A4, and A5. At the end of the learning phase, the experimenter performs 100 tests using the learned parameters *θ*. The user feedback is positive as a successful human-robot collaboration. Moreover, the success rate of human-robot collaboration for each group is recorded.

Equation (5) is used to calculate the success rate of human-robot collaboration:

(5) $$\begin{array}{c} \text{Acc}=\frac{{\text{count}}_{T}}{100}, \end{array}$$

where count_*T*_ indicates the number of successful human-robot collaborations during the test.

To explore the success rate of human-robot collaboration, ten people are divided into groups B and C, with two people in group B and eight people in group C. The experimenters in group B are divided into B1 and B2, with one experimenter in each group; the experimenters in group C are divided into C1, C2, C3, and C4, with two experimenters in each group. First, the robot learns the habits of the experimenters in group B 800 times, and then, we switch the users to the experimenters in group C. The experimenters in group C1 perform 100 tests without learning; the experimenters in group C2 perform 100 tests after 400 times of learning; the experimenters in group C3 perform 100 tests after 600 times of learning; and the experimenters in group C4 perform 100 tests after 800 times of learning. This process is adopted to simulate the robot's performance in an actual situation when it is confronted with a new user with different habits.

#### 4.3.3. Comparison of Multimodal Reinforcement Learning Intention Understanding Algorithms with Naive Bayesian Intention Understanding Algorithms

The core of the MRLC framework lies in the multimodal reinforcement learning intentional understanding algorithm, by which the MRLC framework can learn the user's habits. Therefore, the multimodal reinforcement learning comprehension algorithm is compared with the classical traditional intention intentional understanding algorithm (the naive Bayes intention understanding algorithm) in this section. Bayesian decision formula (6) is employed to calculate the probability of each intention when the user expresses the intention, and the intention with the highest probability is taken as the final result:

(6) $$\begin{array}{c} w_{j}|X=\frac{P\left(X|w_{j}\right)P\left(w_{j}\right)}{\sum_{n=1}^{N}P\left(X|w_{n}\right)P\left(\omega_{n}\right)}=\;\frac{\prod_{k=1}^{M}P\left(x_{k}|w_{j}\right)\;P\left(w_{j}\right)}{\sum_{n=1}^{N}\prod_{k=1}^{M}P\left(x_{k}|w_{n}\right)\;P\left(\omega_{n}\right)}, \end{array}$$

where *w*_*j*_ represents the results of intention understanding, and **X** refers to the user input multimodal data matrix, and *x*_*k*_ represents the kth component of **X**.

Meanwhile, the MRLC framework was used to let the robot learn the user's habit from scratch. Ten experimenters were divided into five groups: D1, D2, D3, D4, and D5, each group of two. One experimenter User #1 was randomly selected from each group to collaborate with the robot using the multimodal reinforcement learning intention understanding algorithm. Starting from a learning count of 300, the robot paused learning every 200 times and tested the accuracy of the multimodal reinforcement learning intention understanding algorithm and the naive Bayes intention understanding algorithm 100 times, for a total of three times. After the 700 learning of User #1 was finished, the robot was replaced by another experimenter from the same group, User #2, to collaborate with the robot. This process stimulated the realization of a real scenario where the robot is confronted with a new user with different habits. The second experimenter was the same as the first one, starting from the 300th learning, pausing learning every 200 times, and performing 100 tests, for a total of three tests.

#### 4.3.4. Evaluating MRLC with Human Factors

The degree of success of human-robot collaboration depends on the joint consideration of the robot factor (RF) and the human factor (HF) [34]. The experiment mentioned above is an evaluation of the robot factor. Therefore, this section uses four human factors indicators to evaluate the MRLC qualitatively. The four indicators are trust, anxiety, safety perception, and fatigue [34]. The score interval of each indicator is [1–10], and the lower score means the worse performance under the indicator, 0 means very poor, and 10 means very good. We collected the subjective feelings of all experimenters during the experiment through a questionnaire. It should be noted that during this experiment, we collected the subjective feelings of experimenters after MRLC fully learned the experimenter's habits.

### 4.4. Experiment Results

#### 4.4.1. Differences in User Habits When Expressing Intentions

**Figure 8:**
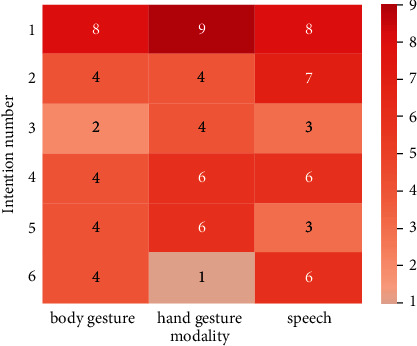
Maximum number of identical expressions with the same intention in the same modality.

The data collected from the questionnaires in Section 4.3.1 were organized in the heat map (Figure 8), with a total of 10 questionnaires received. The maximum number of identical expressions in the same modality was recorded under the same intention. For example, in intention #1, if 7 questionnaires were selected to use one outcome under the modality of body gestures, the value for that position was 7.

Figure 8 reveals that users can reach a consensus on expression habits for specific intentions. For instance, the number of experimenters who choose to use the same expression reaches 8–9 in intention #1, implying that most users tend to express intentions in the same expression.

However, the expression habits vary significantly among users for some intentions, such as intention #6 and intention #3. Since traditional intention understanding algorithms, such as SVM and naive Bayes, aim at demonstrating correlations between modal data and intentions, this phenomenon can tremendously degrade the performance of traditional intention understanding algorithms.

#### 4.4.2. Human-Robot Collaboration Success Rate

**Figure 9:**
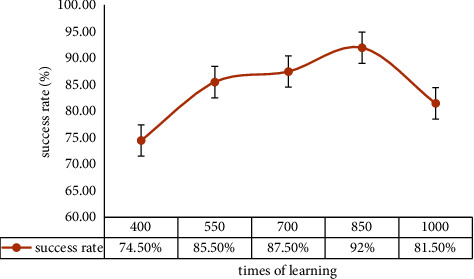
Success rate with the change in learning times.

The data during the experiment are recorded in Table 4, involving the number of failed MRLC algorithm human-robot collaboration trials for individual experimenter of each group. Furthermore, each group's success rate of human-robot collaboration is plotted in Figure 9.

As observed in Table 4, the failure number of human-robot collaboration gradually decreases as learning times increase when learning times are between 400 and 850. The result reflects that the robot is gradually learning the user User #2's habits. Meanwhile, the intention understanding is gradually becoming more accurate. However, the failure number of human-robot collaboration increases when learning times rise to 1000. The principal reason is that the overfitting of the MRLC algorithm leads to a decrease in the effectiveness of human-robot collaboration.

A similar conclusion can be drawn from Figure 9. The success rate of human-robot collaboration in groups A1, A2, A3, and A4 rises and finally reaches 92%. The human-robot collaboration system achieves acceptable levels.

Table 5 presents the change in the failure number of human-robot collaboration with learning after changing experimenters with different C groups of habits.

**Figure 10:**
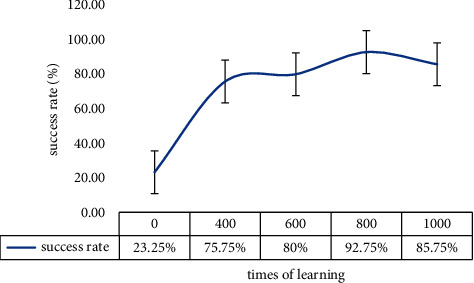
Variation in the human-robot collaboration success rate with the change in learning times when facing new users.

Table 5 and Figure 10 demonstrate a significant decrease in the success rate of human-robot collaboration when the robot faces a user with different habits. In other words, the robot cannot effectively understand the user's intention when facing a new user, which is consistent with our expectation. The success rate of human-robot collaboration increases as learning times increase in the interval of 400 to 800 times of learning, which suggesting that the robot is continuously adapting to new user's habits. The robot reaches a success rate of 93% after 800 times of learning.

As revealed in the previous experiment, the success rate of human-robot collaboration decreases to a specific level when the number of learning times reaches 1000, which is ascribed to the overfitting of MRLC.

This experiment implies that the MRLC framework is malleable and achieves a success rate of more than 90% after hundreds of learning times for a new user's habit.

#### 4.4.3. Comparison of Multimodal Reinforcement Learning Intention Understanding Algorithms with Naive Bayesian Intention Understanding Algorithms

**Figure 11:**
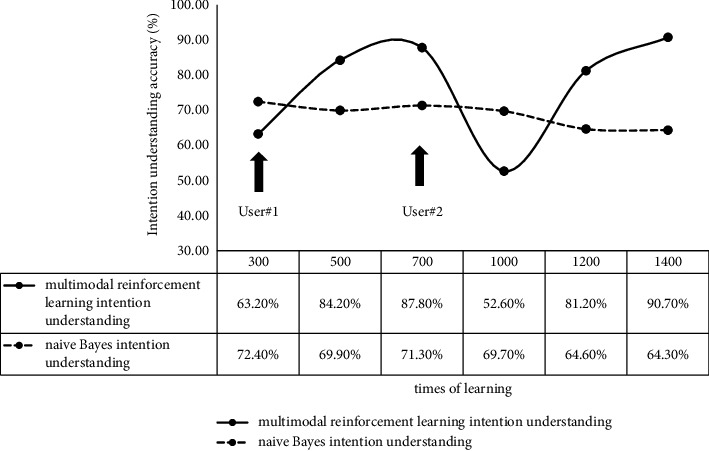
The comparison of accuracy variation of two algorithms with the change in learning times.

As illustrated in Figure 11, the multimodal reinforcement learning intention understanding algorithm achieves 63.2% accuracy of correct intention understanding after 300 times of learning, which is 9.2% lower than the naive Bayes algorithm. However, after 500 times of learning, the multimodal reinforcement learning intention understanding algorithm reaches 84.2% accuracy of intention recognition compared to the other method, with 14.3% performance improvement. The performance difference reaches 16.5% after 700 times of learning.

After changing experimenter User #2, the accuracy of the multimodal reinforcement learning intention understanding algorithm decreases to 52.6% which is lower than 69.7% accuracy of the naive Bayes algorithm. This is induced by the difference in habits between the experimenters. With the increasing learning times, 90.7% accuracy is reached after 700 times of learning, which is significantly higher than 64.3% accuracy of the naive Bayes algorithm.

This experiment demonstrates that the multimodal reinforcement learning intention understanding algorithm eliminates the inconsistent performance of traditional intention understanding algorithms for different users by providing accuracy over 90% after enough times of learning.

#### 4.4.4. Evaluating MRLC with Human Factors

**Figure 12:**
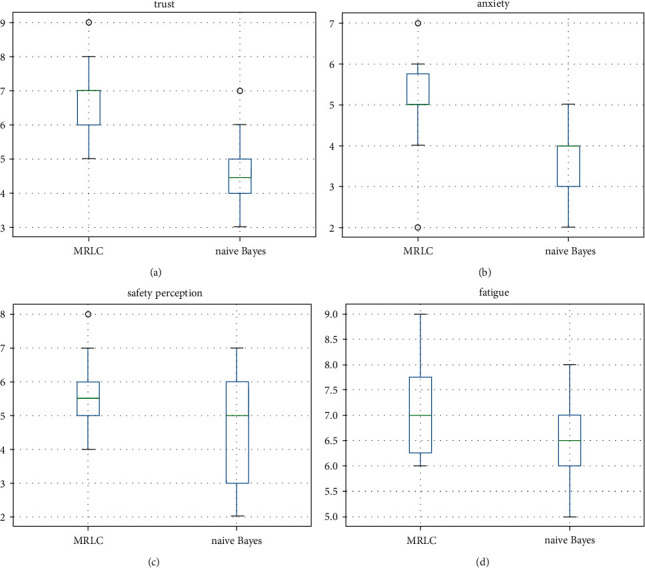
Human factors questionnaire results. (a) Trust; (b) anxiety; (c) safety perception; (d) fatigue.

Ten questionnaires are collected in this article.  Figure 12 illustrates the results of the assessment of human factors indicators for both frameworks. In the indicator of “trust,” MRLC performed significantly better, and most of the experimenters felt that the MRLC collaboration framework brought them more trust. There is not much difference between the two on “anxiety,” with MRLC performing slightly better and a significant portion of the experimenters scoring within 2 points. Neither a collaborative framework was effective in reducing user anxiety with the robot. Similar conclusions are found for the two indicators of safety perception and fatigue. Considering the differences between the experimenters and the resulting errors, there is no significant difference between the two algorithms.

## 5. Conclusion

Most of the traditional human-robot collaboration frameworks specify the process of human-robot collaboration, which corresponds to the user's instructions and the robot's actions.

The MRLC framework has the following advantages over traditional human-robot collaboration frameworks: (1) greater flexibility and higher adaptability. The multimodal reinforcement learning intention understanding algorithm achieves intention understanding in human-robot collaboration and thus solves the problem that the efficiency of traditional human-robot collaboration frameworks decreases when facing different user habits. (2) Stronger reusability. The MRLC framework can be easily applied to other human-robot collaboration scenarios. With the addition of reinforcement learning algorithms, users do not need to make an extra effort on editing the rules of human-robot collaboration. Concurrently, users can directly modify the database between layers to achieve dynamic human-robot task assignments owing to the hierarchical design of the task assignment module.

The experiments suggest that the success rate of collaboration in the MRLC framework reaches more than 90% after many times of learning, which improves over 10% compared with the traditional algorithm.

In our experiments, users are required to learn more than 800 times to achieve an excellent synergistic effect. Since the MRLC framework is based on deep reinforcement learning, it also inherits the shortcomings of deep reinforcement learning algorithms, such as slow convergence.

Therefore, the issues of slow learning speed and slow convergence of the MRLC framework should be overcome in the following research.

## Figures and Tables

**Algorithm 1:**
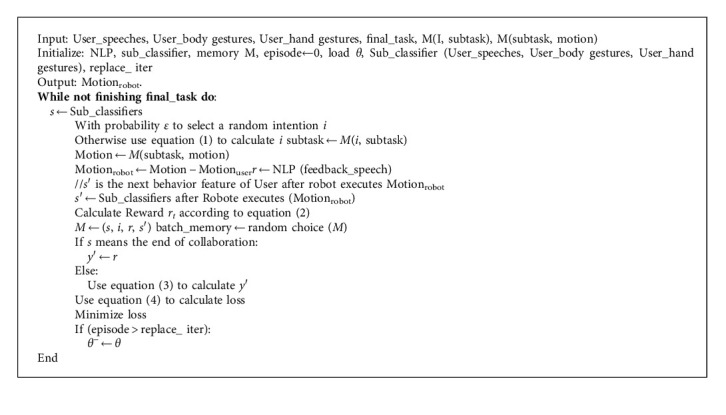
MRLC Multimodal Reinforcement Learning Cooperation

**Table 1:** User's keywords and category numbers.

Keywords	Category numbers
Stop	1
Take a block	2
Put the block on	3
Hand me the block	4
Take the block	5
Put the block aside	6
There is your block	7

**Table 2:** Intentions and numbers.

Intentions	Numbers
The robot stops immediately	1
Robot takes a block and gives it to the user	2
Robot actively picks up a block	3
The robot takes the blocks from the user	4
Putting aside the blocks that the robot clips up	5
Put the blocks that the robot clips up onto the Jenga tower	6

**Table 3:** The questionnaire.

Intentions	Body gestures that can express the intention (if none, fill in 0)	Hand gestures that can express that intention (if none, fill in 0)	Keywords that can express the intention (if none, fill in 0)
The robot stops immediately			
The robot takes a block and gives it to the user			
The robot actively picks up a block			
The robot takes the block from the user			
Put aside the block that the robot clips up			
Put the blocks that the robot clips up onto the Jenga tower			

**Table 4:** Number of failures for different experimenters' human-robot collaboration results.

Times of learning/group	User #1	User #2
400/A1	30	21
550/A2	13	16
700/A3	12	13
850/A4	9	7
1000/A5	20	17

**Table 5:** The failure numbers of human-robot collaboration with the change in learning times when facing new users.

Times of learning/Group	User #1	User #2
0/C1	75.5	78
400/C2	27	21.5
600/C3	21	19
800/C4	8.5	6
1000/C5	17	11.5

## Data Availability

The data used to support the findings of this study are available from the corresponding author upon request.
